# Materials and Device Designs for Wireless Monitoring of Temperature and Thermal Transport Properties of Wound Beds during Healing

**DOI:** 10.1002/adhm.202302797

**Published:** 2023-11-29

**Authors:** Hanjun Ryu, Joseph W. Song, Haiwen Luan, Youngmin Sim, Sung Soo Kwak, Hokyung Jang, Young Jin Jo, Hong‐Joon Yoon, Hyoyoung Jeong, Jaeho Shin, Do Yun Park, Kyeongha Kwon, Guillermo Antonio Ameer, John A. Rogers

**Affiliations:** ^1^ Department of Advanced Materials Engineering Chung‐Ang University Anseong 17546 Republic of Korea; ^2^ Department of Intelligence Energy and Industry Chung‐Ang University Seoul 06974 Republic of Korea; ^3^ Department of Biomedical Engineering Northwestern University Evanston IL 60208 USA; ^4^ Center for Advanced Regenerative Engineering Northwestern University Evanston IL 60208 USA; ^5^ Querrey Simpson Institute for Bioelectronics Northwestern University Evanston IL 60208 USA; ^6^ School of Electrical Engineering Korea Advanced Institute of Science and Technology Daejeon 34141 Republic of Korea; ^7^ Center for Bionics of Biomedical Research Institute Korea Institute of Science and Technology Seoul 02456 Republic of Korea; ^8^ Science Corp. 1010 Atlantic Ave. 100 Alameda CA 94501 USA; ^9^ Department of Electronic Engineering Gachon University Seongnam 13120 Republic of Korea; ^10^ Department of Electrical and Computer Engineering University of California Davis, Davis CA 95616 USA; ^11^ Department of Biomedical Engineering Northwestern University Evanston IL 60208 USA; ^12^ Center for Advanced Regenerative Engineering Northwestern University Evanston IL 60208 USA; ^13^ Department of Surgery, Feinberg School of Medicine Northwestern University Chicago IL 60611 USA; ^14^ Querrey Simpson Institute for Bioelectronics Northwestern University Evanston IL 60208 USA; ^15^ Chemistry of Life Processes Institute Northwestern University Evanston IL 60208 USA; ^16^ International Institute for Nanotechnology Northwestern University Evanston IL 60208 USA; ^17^ Simpson Querrey Institute for Bionanotechnology Evanston IL 60208 USA; ^18^ Department of Mechanical Engineering Northwestern University Evanston IL 60208 USA; ^19^ Department of Materials Science and Engineering Northwestern University Evanston IL 60208 USA; ^20^ Department of Neurological Surgery, Feinberg School of Medicine Northwestern University Evanston IL 60208 USA; ^21^ Department of Mechanical and Aerospace Engineering University of California, San Diego La Jolla CA 92093 USA

**Keywords:** bioresorbable sensor, chronic wound, temperature sensor, thermal conductivity sensor, wireless platform

## Abstract

Chronic wounds represent a major health risk for diabetic patients. Regeneration of such wounds requires regular medical treatments over periods that can extend for several months or more. Schemes for monitoring the healing process can provide important feedback to the patient and caregiver. Although qualitative indicators such as malodor or fever can provide some indirect information, quantitative measurements of the wound bed have the potential to yield important insights. The work presented here introduces materials and engineering designs for a wireless system that captures spatio‐temporal temperature and thermal transport information across the wound continuously throughout the healing process. Systematic experimental and computational studies establish the materials aspects and basic capabilities of this technology. In vivo studies reveal that both the temperature and the changes in this quantity offer information on wound status, with indications of initial exothermic reactions and mechanisms of scar tissue formation. Bioresorbable materials serve as the foundations for versions of this device that create possibilities for monitoring on and within the wound site, in a way that bypasses the risks of physical removal.

## Graphical abstract

Temperature, thermal conductivity‐based wound monitoring system using a wireless communication platform provides continuously wound healing status for diabetic patients. Multi‐arrayed temperature sensors and a thermal actuator facilitate spatio‐temporal temperature maps across the wound areas to track the wound‐healing process. In vivo studies demonstrate insights determined by real‐time, long‐term wound status monitoring such as the end of exothermic reactions and formation of scar tissue.

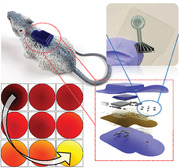

## 1 Introduction

The skin, composed of the epidermis, dermis, and subcutaneous layers, is the largest organ of the human body. Skin serves many functions, as a protective barrier against hazardous substances, as a mechanism to aid in homeostasis, and as a physical sensory interface to the environment.^[^ ^1^ ^]^ Damage to the skin can lead to infections and associated health risks, especially for patients in intensive care units or other vulnerable populations.^[^ ^2^ ^]^ For example, individuals with diabetic mellitus lack the ability to regulate blood glucose levels, which leads to disruptions in normal blood circulation and wound healing processes.^[^ ^3^ ^]^ Such effects create susceptibility to formation of chronic wounds (e.g., diabetic foot ulcers), with healing periods that extend over months even with regular medical care. Regeneration of these and other chronic wounds such as pressure and venous ulcers is a major health challenge that affects between 2.4 and 4.5 million people, amounting to hundreds of billions of dollars annually for medical costs in the United States.^[^ ^4^ ^]^ Treatments based on medications,^[^ ^5^ ^]^ wound dressings,^[^ ^6^ ^]^ cell transplantations,^[^ ^7^ ^]^ negative pressure therapies,^[^ ^8^ ^]^ and electrotherapies^[^ ^9^ ^]^ are effective in promoting the natural processes of wound‐healing, but regular outpatient treatments require careful management tailored according to wound status, often conducted by healthcare professionals. Traditional qualitative wound conditions such as purulent exudate, erythema, edema, pain, warmth, or malodor can reflect general aspects of status, but do not serve as a precise quantification system for automated alerts to patients or caregivers.^[^ ^10^ ^]^

Physiochemical factors such as temperature, pH, humidity, and enzymes are essential parameters related to healing.^[^ ^11^, ^12^ ^]^ The pH of a wound is a simple, but important, indicator of wound condition, with trends from alkaline to neutral as the wound heals.^[^ ^13^ ^]^ Abnormal values of wound pH typically indicate infections.^[^ ^14^ ^]^ Uric acid and lactate are other biomarkers of healing status and infection.^[^ ^15^, ^16^ ^]^ Reported schemes for measuring the concentration of uric acid rely on changes in electrochemical potential, but with limitations due to confounding influences of humidity, temperature, biofouling, and other effects. A simple physiochemical indicator that avoids these complications is wound temperature, typically measured at healthy skin adjacent to the site of the wound, as in the clinical signs and symptoms checklist.^[^ ^17^ ^]^ Cell proliferation, tissue remodeling, and inflammatory responses contribute to exothermic reactions during the healing process.^[^ ^18^, ^19^, ^20^ ^]^ Fibroblasts, for example, produce collagen and extracellular matrix components by consuming energy and releasing heat as a byproduct. The formation of granulation tissue to fill the wound also generates heat during the healing process. Each of these exothermic reactions mainly occurs near the wound site. Smart bandages support wireless temperature and impedance monitoring,^[^ ^21^ ^]^ but without spatial resolution that is often essential, given the heterogeneous nature of most wounds.^[^ ^22^ ^]^ Variabilities in wound depth, shape, and degree of contamination demand segmented approaches in monitoring and care. Thermal imaging technology can provide wound temperature information from 25.1–35.3 °C depending on wound type and patients with a sensitivity of ≈0.1 °C,^[^ ^23^, ^24^ ^]^ but this measurement approach requires optical access to the wound. Arrays of temperature sensors and thermal actuators with real‐time, wireless data communication interfaces might have utility in this context, for spatio‐temporal mapping of not only temperature but also thermal conductivity, the latter as a surrogate for hydration status and/or perfusion. Compared to normal skin and scar tissue, wound tissue saturated with biofluid exhibits a higher thermal conductivity,^[^ ^27^, ^28^, ^29^ ^]^ a parameter detectable by thermal sensors and actuators, further emphasizing their potential for comprehensive spatio‐temporal mapping.

Here, we report the materials and engineering principles for a system of this type, with a simple graphical user interface and wireless link to a smartphone. The technology returns spatial maps of changes in temperature and thermal conductivity across the area of a wound, referenced to corresponding properties of healthy skin at an adjacent location, continuously throughout the healing process. Finite element analysis (FEA) simulations confirm the mechanical flexibility of the system and the minimal strains that occur at its interface with the wound. Animal experiments demonstrate that data collected with technologies of this type can accurately track the wound‐healing process and the formation of scar tissue. Studies using a diabetic animal model suggest some promise for this type of monitoring in the context of chronic wounds. Additional results illustrate the potential for use of bioresorbable temperature sensors in this context, as a strategy to avoid complications that could otherwise arise from removal of devices from the wound site. Collectively, these findings offer a simple but powerful option for monitoring the processes of wound healing, with potential to improve patient care.

## 2 Results and Discussion

**Figure 1:**
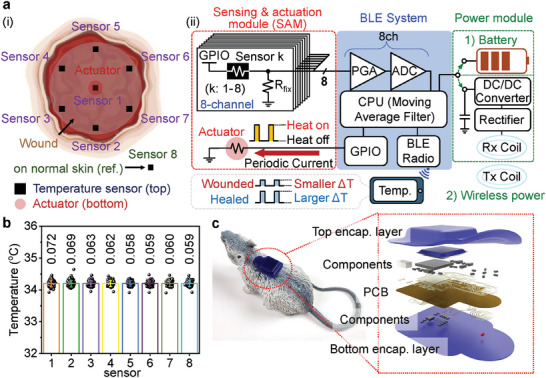
Schematic illustrations and diagrams of real‐time wound monitoring systems. a) Schematic illustrations of positions of temperature sensors and a thermal actuator on a wound (left), and operational diagram of the system (right). GPIO, general‐purpose input/output; PGA, programmable gain amplifier; ADC, analog‐to‐digital converter; CPU, central processing unit; BLE, Bluetooth low energy. b) Temperature measurement results from eight different sensors after calibration. The average standard deviation of temperature is 0.063 °C. c) Photograph of a WMS on a toy mouse and an exploded‐view illustration of the WMS. PCB, printed circuit board.

**Figure** **1**a shows a schematic illustration of a collection of sensors and an actuator over a wound and a simplified block diagram of the entire system. Seven temperature sensors (marked as “sensor 1–7” in Figure 1a‐(i) in a radial arrangement (one central and others 3 mm radius, periodic distributed at 60° intervals) span the area of a wound; an additional separate temperature sensor (marked as “sensor 8” in Figure 1a‐i) interfaces with healthy skin. A thermal actuator (i.e., a heater, marked as “actuator” in Figure 1a‐i) lies on the opposite side of the central temperature sensor, where the wound likely recovers last (Figure S1, Supporting Information). The entire wound monitoring system (WMS) consists of three parts: a sensing and actuation module, a Bluetooth low energy (BLE) system on a chip (SoC), and a power module. Various design layouts appear in Figures S2–S5 (Supporting Information). The module for sensing and actuation consists of negative temperature coefficient thermistors (10 kΩ ± 1%) and fixed resistors (11.3 kΩ ± 1%) for temperature sensing, and a fixed resistor (11.3 kΩ ± 1%) for thermal actuation. Each thermistor and resistor comprises eight voltage dividers that measure temperature with a resolution of 0.02 °C. Figures S6 and S7 (Supporting Information) demonstrate heating behavior with/without an encapsulation layer using various resistors (200–900 Ω). For these conditions, after heating for 15 s with resistors of 700–900 Ω, the temperature is less than 40 °C. Experiments in the following use a 800 Ω resistor to ensure that the temperature remains below safety thresholds.^[^ ^28^ ^]^ The BLE SoC includes an 8‐channel programmable gain amplifier (PGA), an 8‐channel 12 bit analog to digital converter (ADC), a central processing unit (CPU), a general‐purpose input/output (GPIO), and a BLE antenna (Table S1, Supporting Information). Two GPIOs independently power the temperature sensors and the thermal actuator with different duty cycles to minimize power consumption. For in vivo experiments, recordings from the temperature sensors occur once per minute and once per second when the thermal actuator is off and on, respectively. The thermal actuator operates once per 3 h. A smartphone user interface graphically displays the results of measurements of temperature in real‐time, with options in magnification and auto‐save functions (Figure S8, Supporting Information). Systematic studies indicate the ability to measure temperature with a standard deviation of ±0.06 °C (Figure 1b), which is sufficient for present purposes.^[^ ^29^ ^]^ Options in power supply include a small battery and a system for wireless power transfer by electromagnetic induction. The battery supports operation for several weeks, but involves some bulk and weight that must be considered in studies with small animal models. The wireless powering option avoids these disadvantages, but only offers operation in a restricted space adjacent to a radio frequency transmission system. Figure 1c shows a photograph of WMS on a mouse and an exploded‐view illustration.

**Figure 2:**
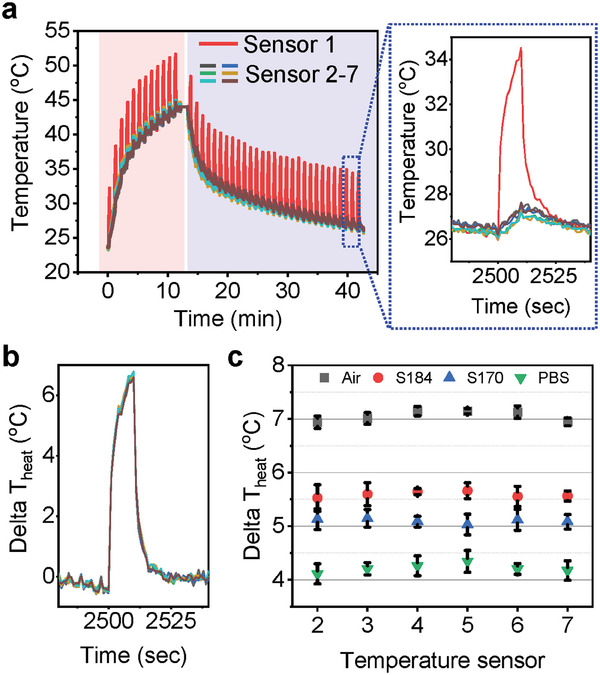
Evaluation of temperature sensors. a) Wireless temperature measurements at various temperatures in an oven (red area) and at room temperature (blue area). The inset graph is a magnified example. b) An example of temperature differences between sensor 1 and sensors 2–7. c) Maximum Δ*T*_heat_ for each sensor depending on environmental conditions.

Environmental and physical conditions affect the transport of heat from the thermal actuator across the wound site.^[^ ^25^, ^30^, ^31^ ^]^ The electronics themselves, specifically the interconnect traces that connect to the two ends of the actuator, also affect thermal diffusion, as illustrated in the distributions of temperature near the thermal actuator (Figure S9, Supporting Information). Spiral‐shaped arrangements of interconnect traces around the actuator enhance the uniformity of the heat distribution (Figure S9c, Supporting Information) compared to the case of linear‐shaped interconnect traces (Figure S9d, Supporting Information). **Figure** **2** summarizes the dependence of the response of the system on environmental temperature and thermal conductivity. The thermal actuator operates in 1 min intervals from room temperature (23 °C) to 45 °C (Figure 2a). The WMS undergoes passive cooling in ambient air conditions. “Sensor 1” measures transient increases in temperature with a magnitude of ≈8 °C during the operation of the thermal actuator. The other sensors (sensors 2–7) indicate increases of ≈1 °C. The differences in temperature (i.e., Δ*T*_heat_) between the central (sensor 1) and peripheral (sensor 2–7) sensors are in Figure 2b. Due to similar environmental conditions, Δ*T*_heat_ is ≈0 °C except during the heating period. The thermal actuator induces Δ*T*_heat_ of 6–7 °C for temperatures across the range of interest, that is, 23 and 45 °C. Slight deviations across the collection of sensors arise from non‐uniformities in the radial temperature distribution (Figure S10, Supporting Information). Air, poly(dimethylsiloxane) (PDMS) (two commercial formulations from Dow Corning, S184 and S170), and Dulbecco's phosphate‐buffered saline (DPBS, pH 7.4) mimic different wound conditions to define a range of values of Δ*T*_heat_ comparable to those expected with wounds (Figure 2c and Figure S11, Supporting Information). Two PDMS formulations (S184 and S170) have thermal transport properties similar to those of dehydrated and hydrated skin, respectively. Most of the sensors have Δ*T*_heat_ of ≈7, 5.5, 5, and 4 °C in air, S184, S170, and DPBS, respectively. Evaluations of long‐term operational stability of the devices rely on immersion in DPBS for >40 h (Figure S12, Supporting Information). The results indicate that the sensors and the thermal actuator operate in these conditions without significant malfunctions. Furthermore, stable temperature monitoring is possible when placed on top of a hydrogel film throughout the course of drying in an open laboratory environment at physiological temperatures, to mimic the transition from a wet wound bed to dry tissue (Figure S13, Supporting Information). These results illustrate that dry and wet wound conditions correspond to high and low values of Δ*T*_heat_, respectively. This parameter thus serves as a basis for estimating the progress of wound healing, where Δ*T*_heat_ increases with extent of healing, consistent with a decrease in the thermal conductivity.

**Figure 3:**
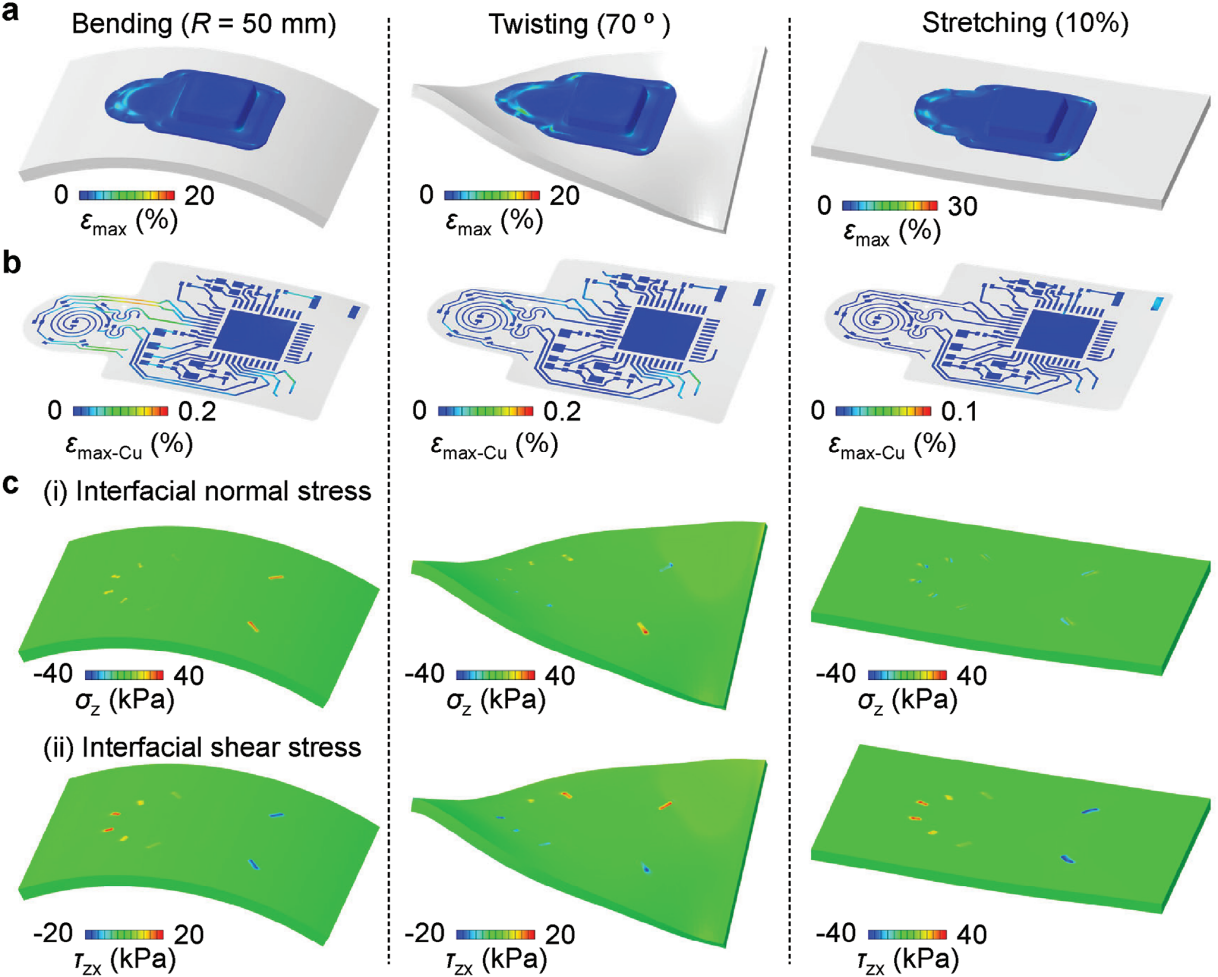
Finite element analysis (FEA) results showing the strain/stress distributions and deformed configurations of an WMS during bending (radius of curvature 50 mm), twisting (70°), and uniaxial stretching (10%), respectively: a) the encapsulated WMS device, b) the copper layer of the electronics, and c) the computed i) normal (*σ_zz_*) and ii) shear (*τ_zx_*) stresses at the device/skin interface.

FEA and mechanical test results quantify the nature of mechanical deformations of the WMS and interfacial stresses that appear between the WMS and skin (**Figure** **3** and Figure S14, Supporting Information). Figure S15 (Supporting Information) shows the location of the WMS relative to the wound site, and configurations that result from bending with a radius of 50 mm along the long axis, twisting through a rotation angle of 70°, and 10% uniaxial stretching. Eight suture points on the edge of the silicone encapsulation layer (Silbione RTV 4420, Elkem) fix the WMS on the skin. The WMS can accommodate bending, twisting, and stretching deformations associated with natural motions at most body locations. The maximum strain across the encapsulation layer during these deformations is less than 30%, within the elastic range of the silicone material (Figure 3a). The copper (Cu) conductive traces on the flexible printed circuit board (fPCB) inside the silicone encapsulation are flexible and remain within an elastic regime. The maximum strain in the Cu layers is ≈0.2%, lower than the yield strain of 0.3% (Figure 3b). Most of the strain in the electronic layers occurs in the interconnects, not at solder joints between the fPCB and electronic components, thereby avoiding delamination of electronic components during mechanical deformations. The soft silicone encapsulation material gives rise to the overall complaint mechanics of the WMS. FEA studies indicate that the interfacial normal and shear stresses are imperceptible (within 20 kPa^[^ ^9^ ^]^) in most areas including the wound location during deformations, except for the suture regions (Figure 3c and Figure S16, Supporting Information).

**Figure 4:**
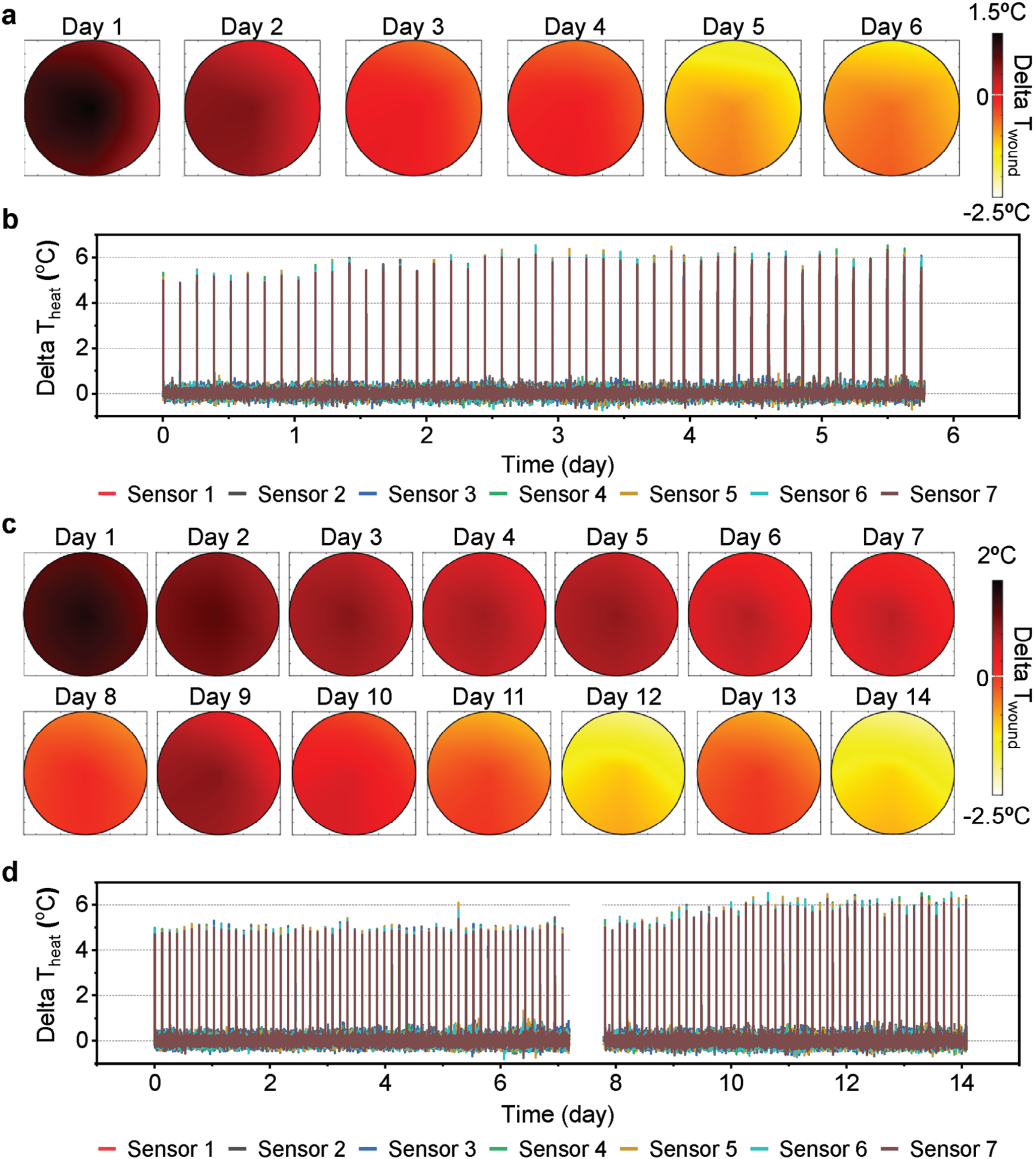
In vivo experimental results obtained using normal mouse models. a) Interpolated Δ*T*_wound_ maps and b) Δ*T*_heat_ of the control group. c) Interpolated Δ*T*_wound_ maps and d) Δ*T*_heat_ of the experimental group. An interruption in the wireless interface caused data loss between day 7 and 8.

Demonstrations of a practical application of the WMS involve measurements of temperature and Δ*T*_heat_ from a non‐wounded normal mouse as a control group and a normal mouse with a full‐thickness excisional dermal wound as an experimental group (**Figure** **4**). The average skin temperature of a mouse is 35 ± 1 °C depending on its health status and level of physical activity (Figures S17 and S18, Supporting Information). Figure 4a shows interpolated maps of temperature differences between “sensors 1–7” and “sensor 8” (i.e., Δ*T*_wound_). Differences of ≈0.5 °C at the beginning of the experiment result from responses of the immune system due to the suturing process to hold the WMS in place. This small value of Δ*T*_wound_ quickly decreases to ≈0 °C due to minimal exothermic reactions. Monitoring over a period of a week indicates that Δ*T*_wound_ is usually lower than 0 °C. Measurements of Δ*T*_heat_ confirm that the value for normal skin is ≈6 °C (Figure 4b). Based on expected relative values of thermal conductivity, a wet wound and scar tissue should exhibit values of Δ*T*_heat_ smaller and larger than 6 °C, respectively. The experimental group shows wound and skin temperatures of 36 and 34 °C, respectively, after surgery (Figure S18, Supporting Information). The wound temperature gradually decreases with time, while the skin temperature varies between 34 to 36 °C. The value of Δ*T*_wound_ also gradually decreases over time and finally reaches a value below 0 °C after 10 days, at the end of exothermic reactions associated with healing (Figure 4c). Since a wound saturated with biofluid should have a thermal conductivity higher than that of normal skin and scar tissue,^[^ ^25^, ^26^, ^27^ ^]^ Δ*T*_heat_ is ≈5 °C, lower than that of normal skin (≈6 °C, Figure 4b,d). The value of Δ*T*_heat_ remains low for ≈9 days and then increases to 6.5 °C after 10 days due to formation of scar and epithelial tissue, correlated with the timescale for wound healing inferred from Δ*T*_wound_. Scar formation in a normal mouse occurs within 7–10 days (Figure S19, Supporting Information). Reconstruction of skin layers requires additional time.

**Figure 5:**
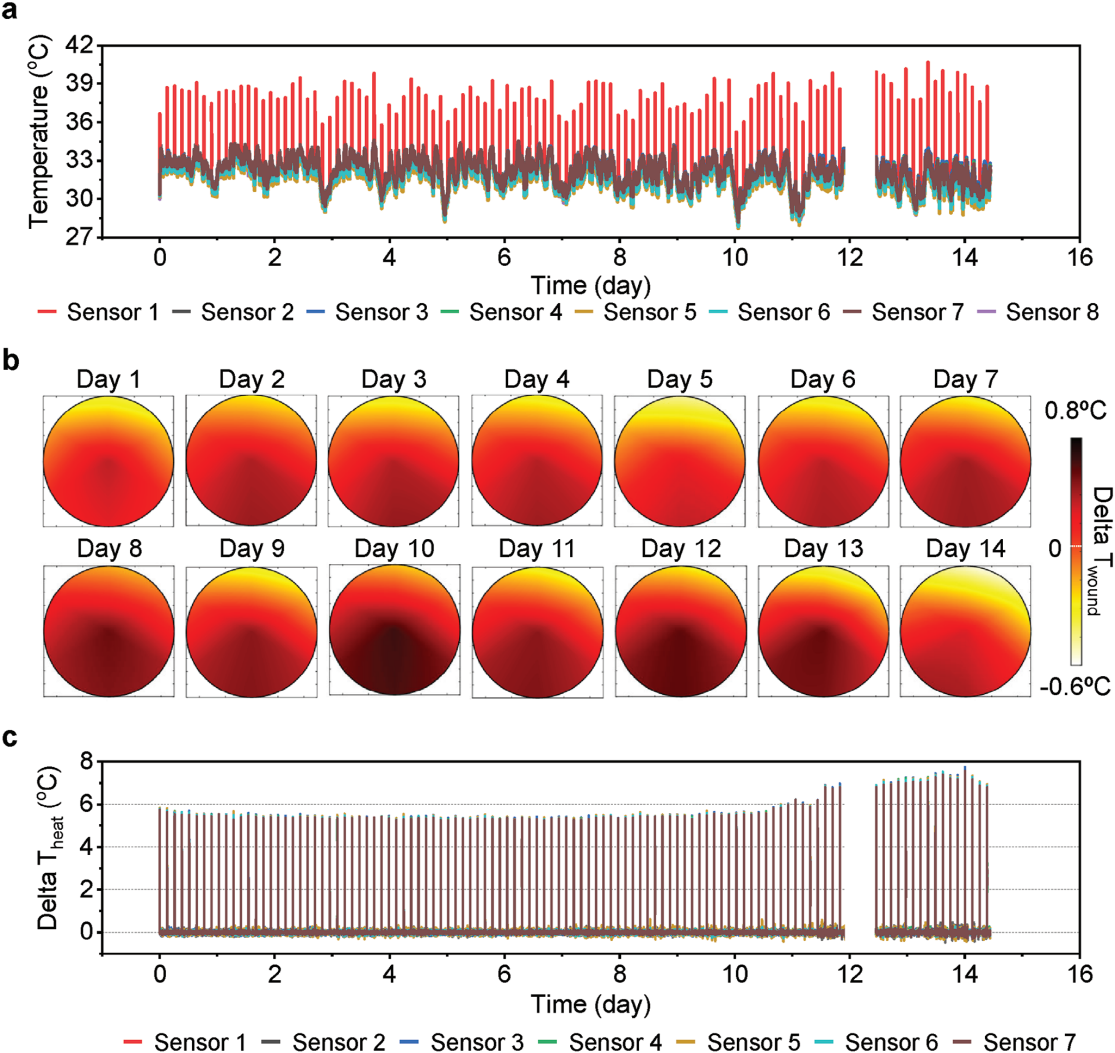
In vivo experimental results obtained from diabetic mouse models. a) Real‐time temperature, b) interpolated Δ*T*_wound_ maps, and c) Δ*T*_heat_ of the experimental group. An interruption in the wireless interface caused data loss between day 12 and 13.

**Figure** **5** demonstrates the system with a diabetic mouse that has a full‐thickness excisional dermal wound. Reduced levels of physical activity and metabolic processes lead to average wound temperatures of only 33 ± 0.5 °C (Figure 5a). Figure S20 (Supporting Information) shows a photograph of a mouse after wearing the WMS, and 1 week interval photographs of the wound to confirm the healing process. The results suggest that the wound dries and forms scar tissues after 2 weeks. Unlike a normal mouse, the wound temperature and the value of Δ*T*_wound_ do not show significant changes over 2 weeks (Figure 5a,b). On the other hand, Figure 5c indicates that Δ*T*_heat_ reflects the time of scar tissue formation. Specifically, Δ*T*_heat_ remains at ≈5.5 °C for 10 days and then increases to ≈7 °C after 12 days, coincident with formation of scar tissues between the wound and the WMS. This increase corresponds to a reduction in thermal conductivity associated with the scar tissue that replaces the moist wound environment. The battery‐less platform offers similar measurement capabilities (Figures S5 and S21, Supporting Information).

**Figure 6:**
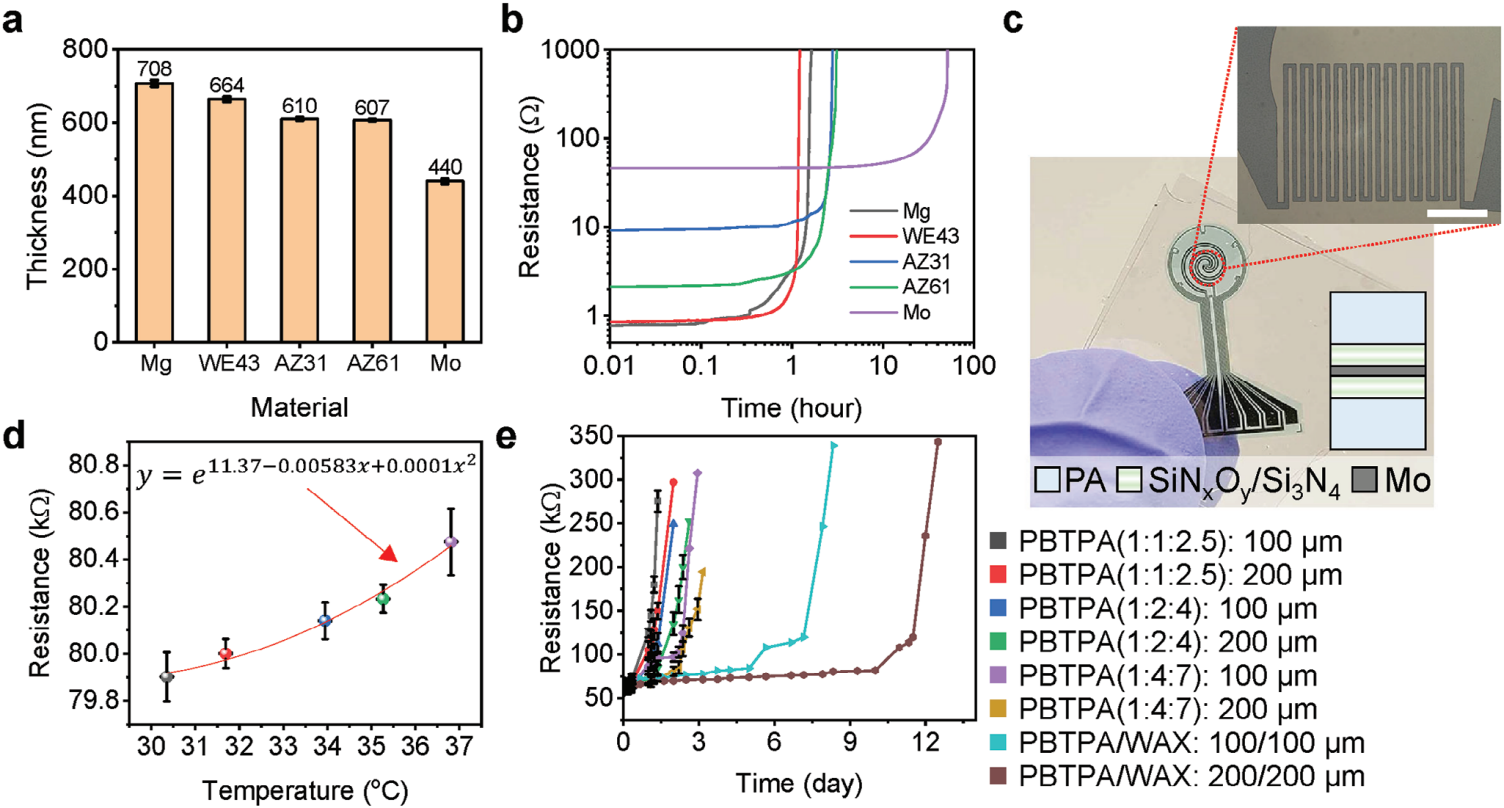
Materials and designs for a bioresorbable temperature sensor. a) Thicknesses of various bioresorbable metal layers formed by electron beam evaporation. b) Changes in the resistance of bioresorbable metals during immersion in DPBS (pH 7.4) at room temperature. c) Optical images of bioresorbable temperature sensors. The magnified image highlights a temperature sensor, scale bar: 100 µm. d) Resistances of bioresorbable temperature sensors as a function of temperature. e) Resistances of bioresorbable temperature sensors with different encapsulation layers as a function of time of immersion in DPBS (pH 7.4) at 37 °C.

Bioresorbable versions of the sensors and actuators reported here represent important options that avoid risks to tissue damage during removal. Here, natural processes of hydrolysis cause the sensors to disappear into the wound bed in a biocompatible manner after a desired functional period. Magnesium (Mg), Mg‐3.5Y‐2.3Nd‐0.5Zr (RE43), Mg‐3.5Al‐1 Zn (AZ31), Mg‐6Al‐1 Zn (AZ61), and molybdenum (Mo) are attractive candidates for conductive elements of such bioresorbable components, in thin, flexible form factors. **Figure** **6**a and Figure S22 (Supporting Information) show layers of these metals deposited by electron beam evaporation on a SiO_2_/Si wafer. The thicknesses of the films of Mg, WE43, AZ31, AZ61, and Mo are 700, 660, 610, 600, and 440 nm, respectively. Figure 6b compares changes in resistance of traces of these metals while immersed in DPBS (pH 7.4) at room temperature. The traces of Mg and Mg alloys lose conductivity in a few hours; those of Mo maintain conductivity over a day. With appropriate encapsulation layers, these metals can be designed to support stable operation in a wound environment over a relevant time period. Figure 6c and Figure S23 (Supporting Information) show examples of bioresorbable temperature sensors using polyanhydride (PA, 1 mm thick), SiN*_x_* O*_y_*/Si_3_N_4_ (multi‐layer, total 5 µm thick), and Mo (23 nm thick for sensors, 223 nm thick for interconnect wires; 2 nm thick for Ti as an adhesion promoter layer). All of these materials are bioresorbable.^[^ ^32^, ^33^, ^34^ ^]^ SiN*_x_* O*_y_*/Si_3_N_4_ and Mo layers lie close to the neutral mechanical plane of a multilayer structure that involves the top and bottom layers of PA.^[^ ^35^ ^]^ Water penetration into the PA and SiN*_x_* O*_y_*/Si_3_N_4_ encapsulation layers lead to dissolution of Mo in DPBS (pH 7.4) at 75 °C (Figure S24, Supporting Information). Figure 6d confirms the operation as a temperature sensor through changes in resistance. Figure 6e demonstrates the ability of additional encapsulation layers to extend the functional lifetimes. Specifically, liquid mixtures of 4‐pentenoic anhydride (4PA), 1,3,5‐triallyl‐1,3,5‐triazine‐2,4,6(1H,3H,5H)‐trione (TTT), and 1,4‐butanedithiol (BDT) can form encapsulation layers of polybutanedithiol 1,3,5‐triallyl‐1,3,5‐triazine‐2,4,6(1H,3H,5H)‐trione pentenoic anhydride (PBTPA).^[^ ^36^ ^]^ Different ratios of 4PA, TTT, and BDT (1:1:2.5, 1:2:4, and 1:4:7) and layers with different thicknesses (100 and 200 µm) demonstrate the trends. The case of a 200 µm thick layer of PBTPA (1:4:7) offers stable resistance for 24 h. An additional encapsulation layer of wax (200 µm thick, mixture of beeswax and candelilla wax, weight ratio 2:3) under the PBTPA layer delays water penetration, thereby increasing the stability for about 300 h; increasing the thicknesses of the sensor metallization (e.g., 23 to 440 nm) increases the lifetime.

## 3 Conclusion

The real‐time spatio‐temporal wound monitoring system introduced here offers capabilities in temperature and thermal transport measurements as an effective means for tracking processes of wound healing. The materials and form factors offer mechanical compatibility during direct contact with the soft tissues of wounds, as supported by simulation results. Temperature differences between the wound and adjacent healthy skin arise from exothermic reactions within the wound, as a clear indicator of expected mechanisms, most pronounced in normal animal models. Thermal transport characteristics provide the basis for tracking formation of scar tissues through changes in near‐surface hydration levels, as a metric of particular value in diabetic animal models. Low power operation (average ≈150 µW, ≈10 mW for heating, Figure S25, Supporting Information) allows for over 2 weeks operation with miniaturized batteries, sufficient for most practical applications on non‐chronic wounds. Wirelessly powered options allow unrestricted lifetimes. Bioresorbable versions avoid the need for physical removal of the devices after the healing is complete. In vitro viability assays and Live/Dead staining studies show no significant differences between the control and the two types of devices (Figure S26, Supporting Information). Additional opportunities involve the use of these devices to study inflammatory responses, bacterial infections, and other adverse effects. Given the critical role of bacteria prevention in wound healing, future studies will aim to integrate materials with antibacterial properties and to establish the ability to detect infection levels as infected wounds often exhibit enhanced temperature variations.^[^ ^37^ ^]^ Further development and regulatory advances have the potential to yield a platform for real‐time, antibacterial, universal wound monitoring in the hospital or in the home.

## Conflict of Interest

The authors declare no conflict of interest.

## Supporting information

Supporting Information

## Data Availability

The data that support the findings of this study are available from the corresponding author upon reasonable request.
