# Supplementary material for: Materials and Device Designs for Wireless Monitoring of Temperature and Thermal Transport Properties of Wound Beds during Healing
Source: Adv Healthc Mater. 2023 Nov 29;13(5):2302797. doi: 10.1002/adhm.202302797 (PMC11468708; doi:10.1002/adhm.202302797)
Supplement: Supplementary file 1 — Supporting Information [file ADHM-13-2302797-s001.pdf]

ADVANCED HEALTHCARE MATERIALS

Supporting Information

for *Adv. Healthcare Mater.*, DOI 10.1002/adhm.202302797

Materials and Device Designs for Wireless Monitoring of Temperature and Thermal Transport Properties of Wound Beds during Healing

Hanjun Ryu, Joseph W. Song, Haiwen Luan, Youngmin Sim, Sung Soo Kwak, Hokyung Jang, Young Jin Jo, Hong-Joon Yoon, Hyoyoung Jeong, Jaeho Shin, Do Yun Park, Kyeongha Kwon, Guillermo Antonio Ameer* and John A. Rogers**

Supporting Information

Materials and device designs for wireless monitoring of temperature and thermal transport properties of wound beds during healing

Hanjun Ryu, Joseph W. Song, Haiwen Luan, Youngmin Sim, Sung Soo Kwak, Hokyung Jang, Young Jin Jo, Hong-Joon Yoon, Hyoyoung Jeong, Jaeho Shin, Do Yun Park, Kyeongha Kwon, Guillermo Antonio Ameer*, John A. Rogers**

Method

Fabrication of wireless WMS

A copper-clad polyimide sheet (AP8535R, Pyralux) with a total thickness of 111μm served as the base for the flexible subtrate. A UV laser system (ProtoLaser U4, LPKF) defined circuit traces on the copper layer by ablating the copper to establish the prototype fPCB. A galvanic pulsed electroplater (Contac S4, LPKF) estatablished electrical connections between the top and bottom copper layers through laser-ablated holes. Outcomes of the prototype fPCB studies provided the basis for designing final configuration, outsorced to the ISO-9001 compliant vendor for final fPCB production. A conductive soldering paste (TS391LTL, Chip Quik) attached off-the-shelf, surface-mount electrical components, including a BLE SoC (nRF52832, Nordic Semiconductors), thermistors (NCP03XH, Murata), a rectifier (BAS40XY, NExperia USA Inc.), a DC-DC converter (LTC3255IDD, Analog Devices Inc.), and an antenna (2450AT18A100, Johanson Technology Inc.), to the fPCB. A polyorganosiloxane elastomer film (Silbione RTV 4420, Elkem) encapsulated the devices to provide a hermetic seal.

Software development environment

BLE development kit board (nRF52 DK, Nordic Semiconductor) enabled software development and software validation for BLE SoC (nRF52832, Nordic Semiconductor). USB-to-5pin cable connection between PC and nRF52 DK facilitated seamless data exchange and on-board programming of BLE SoC, ensuring testing and validation of the developed software. Android application served as the user interface on smartphones. Android's official integrated development environment (Android Studio, Google) provided a standardized approach to designing and building the smartphone application.

Finite element analysis

The 3D finite element analysis (FEA) conducted with commercial software Abaqus provided examination of the mechanical response of devices mounted on skin, including reactions to stretching, bending, and twisting. Four-node composite shell elements were used for the polyimide (PI) thin film and copper traces, and eight-node and ten-node solid elements were used for the electronic components and silicone encapsulation, respectively. PI was modeled as a linear elastic material with elastic modulus of 2.5 GPa and Poisson's ratio of 0.34. Copper was modeled as elastoplastic (without hardening), with elastic modulus of 119 GPa and Poisson's ratio of 0.34, and a yield strain of 0.3%. Electronic components were simplified as molding compound material, characterized by elastic modulus of 25 GPa and Poisson's ratio of 0.3. The Silbione RTV 4420 silicone material and the skin were modeled as incompressible elastomeric materials with Mooney-Rivlin hyperelastic behavior, and elastic moduli of 730 kPa and 130 kPa, respectively. During stretching, displacements corresponding to 10% stretching were applied to the ends of the skin part. During bending, the bottom surface of the skin part was pressed against a rigid cylinder with a 50 mm radius. During twisting, the two ends of the skin phantom were twisted by relatively 70°. The study results included various deformed configurations, along with strain and stress distributions across different levels and types of loading.

Wound healing model

All in vivo studies were approved by the Institutional Animal Care and Use Committee (IACUC) at Northwestern University (protocol IS00018748). Both diabetic (db/db) mice (BKS.Cg-m +/+ Leprdb, #000642; homozygous for Leprdb) and healthy mice (C57/BL6J) were purchased from Jackson Laboratory (Bar Harbor, ME, USA). To prevent the healing process via skin contraction, a splinted excisional wound model has been utilized [38]. As previously

described, paired sterilized doughnut-shaped acrylate splints (10-mm inner diameter; 12-mm outer diameter) (3M, St. Paul, MN) were sutured to the center dorsal sides of the mouse with Vetbond (3M) and 6-0 nylon sutures (Ethicon, Cincinnati, OH) after depilation. A 6-mm circular, full-thickness wound was created in the center of each splinted area. For the groups with a device, the device's sensor part was laminated on the center of the wound, and wireless electronic was placed outside the wound. After suturing the device onto the mice, a transparent sterile occlusive dressing, TegaDermTM (3M), was placed over the wound, the splint, and the device. The wounds were left untreated other than replacing the protective dressing. The device continued monitoring the temperature change, and the smartphone, positioned on top of the mouse cage, recorded the signal up to 2 weeks of post-wounding. Digital images of wounds were taken on days 1, 4, 7, and 10 post-injury for normal mice and days 1, 7, and 14 for diabetic mice.

In vitro biocompatibility test

A mouse fibroblast cell line (L929, ATCC® CCL-1™, ATCC, VA, USA), cultured in associated media (ATCC® 30-2003™, ATCC, VA, USA) according to the manufacturer's instructions, was used to provide cells for biocampatibility testing. The testing setup involved seeding 300,000 cells in a 6-well plate with Transwell inserts (Corning, NY, USA) and placing UV-sterlized transient device, consisting of molybdenum and polyanhydride. For non-transient devices, a thin layer of silicone encapsulation was placed on the Transwell. Ater 96 hours, cell viability in the presense of the sensor was determined using alarmaBlue assay (ThermoFisher, MA, USA) and a live/dead staining kit (Invitrogen, MA, USA) according to the the manufacturer's protocol. Cell viability was assessed through fluorescence measurement and imaging using a Cytation5 (Biotek, VT, USA).

Parameter	Pin Name	Type	Current Value (Range)	Description
GPIO_EN	P0.26	Digital I/O	• HIGH for heat on<br>• LOW for heat off	• General Purpose I/O (GPIO)<br>• Supply a constant voltage (VDD-0.4~VDD) and hence, constant current (max 15mA) to the resistive heater.
GPIO2_EN	P0.26		• HIGH during ADC sampling<br>• LOW otherwise	• General Purpose I/O (GPIO)<br>• Provide a supply voltage (VDD-0.4~VDD) to the sensor module only during ADC sampling for low-power operation.
NRF_SAADC_INPUT_AIN0	P0.02	Analo g Input	-	• Successive Approximation ADC (SAADC) Input<br>• Measure the voltage across the temperature sensor 1-8 and convert it into digital format
NRF_SAADC_INPUT_AIN1	P0.03			
NRF_SAADC_INPUT_AIN2	P0.04			
NRF_SAADC_INPUT_AIN3	P0.05			
NRF_SAADC_INPUT_AIN4	P0.28			
NRF_SAADC_INPUT_AIN5	P0.29			
NRF_SAADC_INPUT_AIN6	P0.30			
NRF_SAADC_INPUT_AIN7	P0.31			
NRFX_SAADC_CONFIG_RE SOLUTION	-	-	12 bit (8, 10, 12, 14)	• ADC bit resolution<br>• 14 bit resolution could be achieved by oversampling
SAMPLE_PERIOD	-	-	5ms (min 0.0305ms)	• ADC sampling period<br>• A 32.768kHz crystal enables minimum sampling period of 0.0305ms.
SAMPLE_NUM	-	-	40	• Number of samples to be obtained after the heater on/off transition has occurred
SAMPLE_PERIOD*SAMPLE _NUM (HEAT_ON_TIME)	-	-	5ms X 40 = 0.2s	• Thermal actuator (heater) "on" time<br>• Thermal actuator (heater) is on for SAMPLE_PERIOD*SAMPLE_N UM every TIMER_PERIOD
TIMER_PERIOD (HEAT_ON_PERIOD)	-	-	1s (min 0.0305ms)	• Thermal actuator (heater) "on" period<br>• Thermal actuator (heater) is on for SAMPLE_PERIOD*SAMPLE_N UM every TIMER_PERIOD
2*SAMPLE_PERIOD (BLE_TRANSFER_PERIOD)	-	-	2s	• BLE data transfer period

Table S1. Summary of parameters.

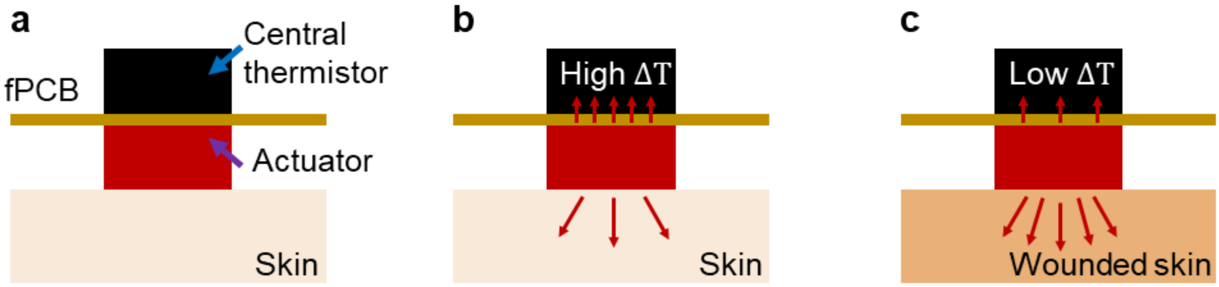

Figure S1. Side view of central sensing and actuating units. (a) Actuator (L×W×H=0.6mm×0.3mm×0.23mm, ceramic-nickel mixture), fPCB (flexible film (AP8535R, Pyralux, DuPont) of copper/PI/copper (thicknesses of 18µm, 75µm and 18µm)), and central thermistor(L×W×H=0.6mm×0.3mm×0.3mm, ceramic) are layered above the skin. (b) Healthy skin, which has low thermal conductivity, transports heat less efficiently, thereby resulting in a higher temperature change at the location of the actuator. (c) Wound tissue, on the other hand, has a higher thermal conductivity due to the presence of biofluids, which leads to a reduced temperature change ΔT.

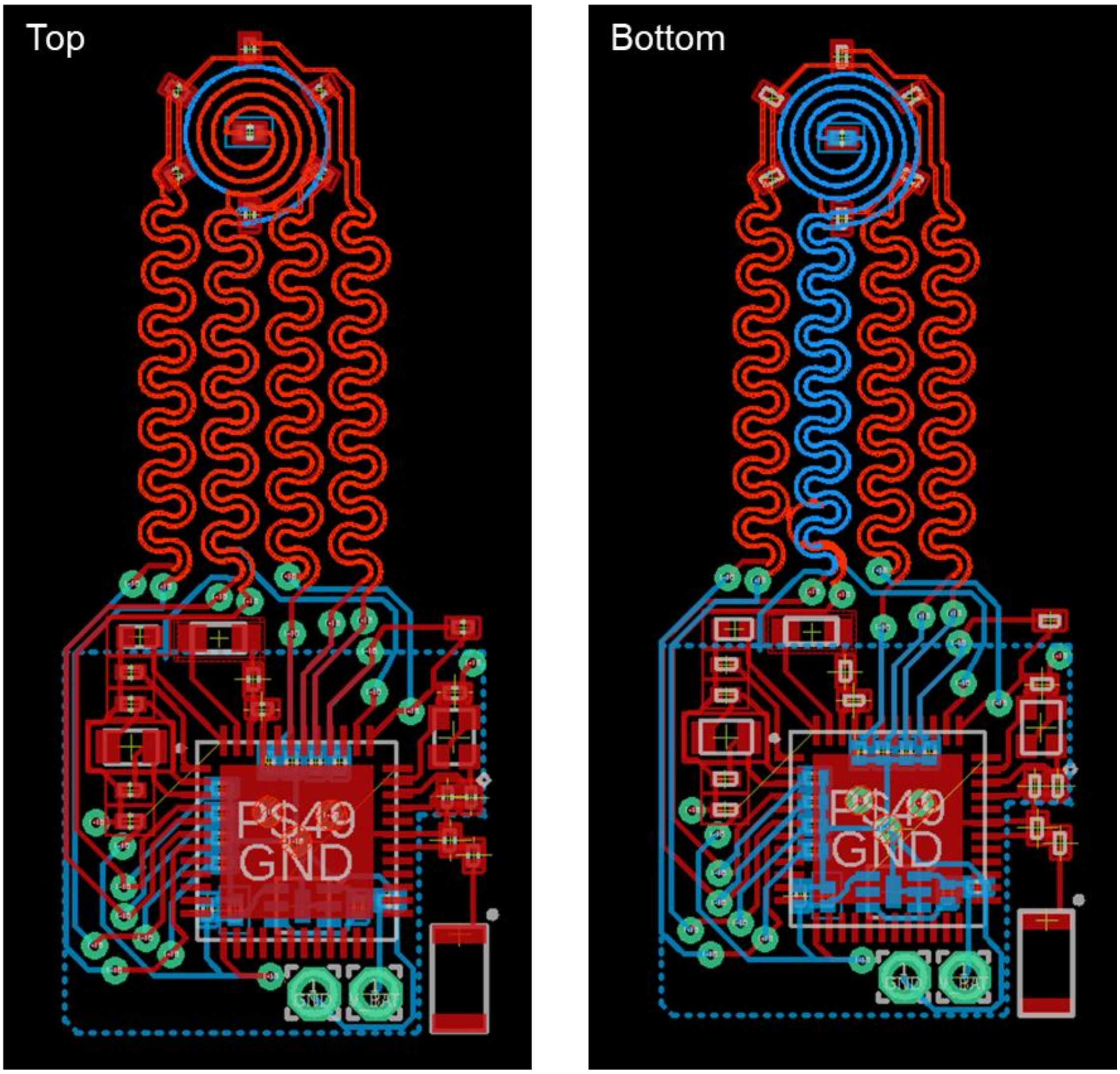

Figure S2. Board outline of the wound monitoring system (long neck version).

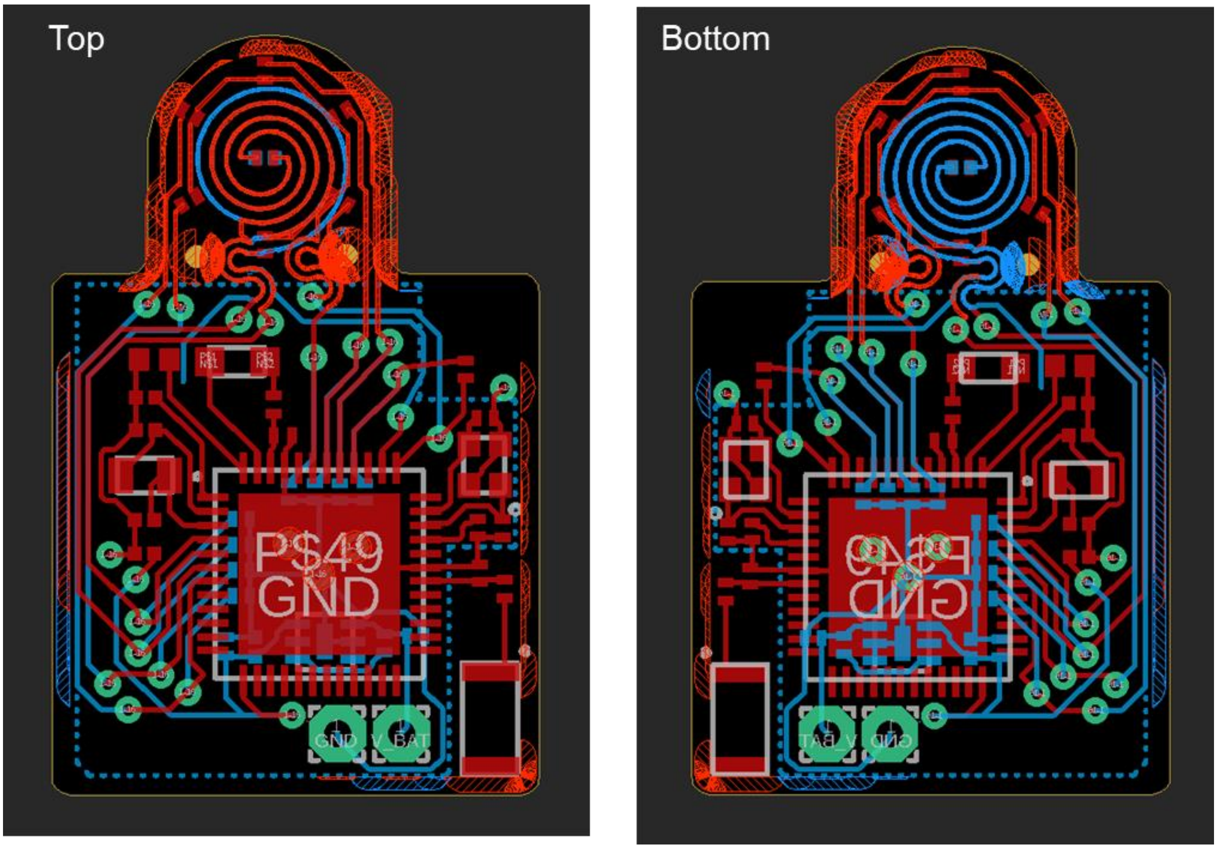

Figure S3. Board outline of the wound monitoring system (short neck version).

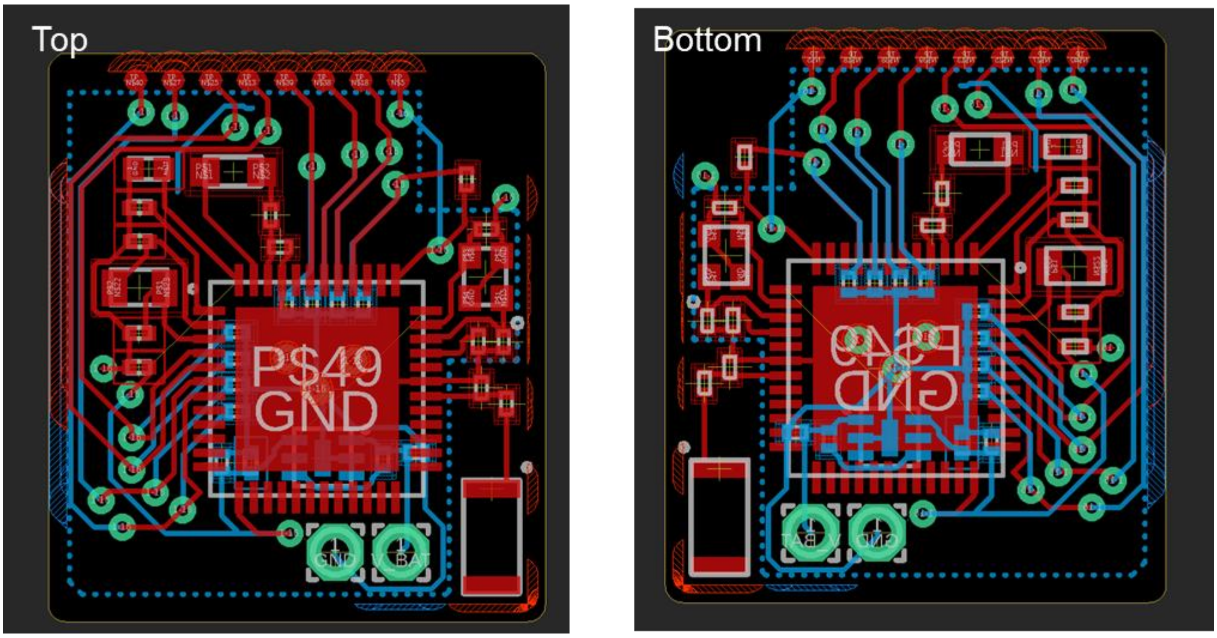

Figure S4. Board outline of the wound monitoring system (bioresorbable sensor version).

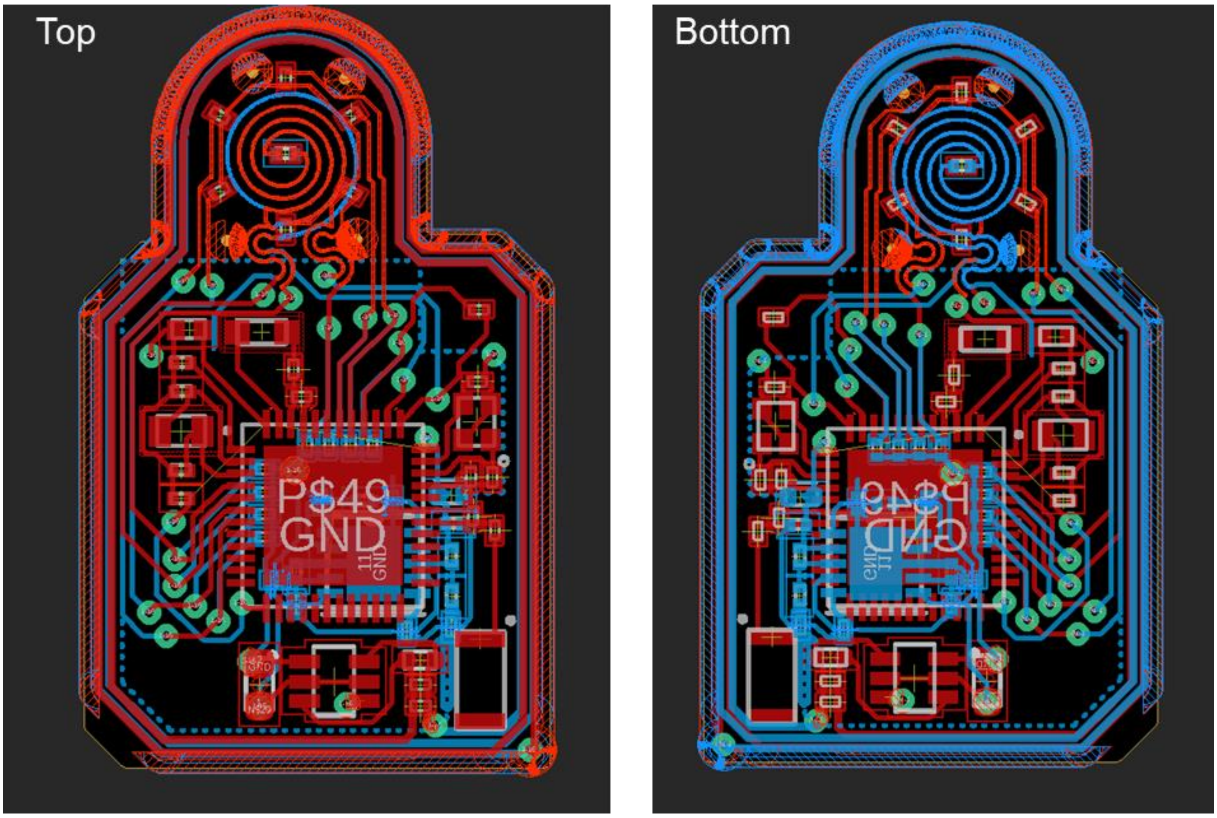

Figure S5. Board outline of the wound monitoring system with Near-Field Communication (NFC) technology for wireless charging (NFC version).

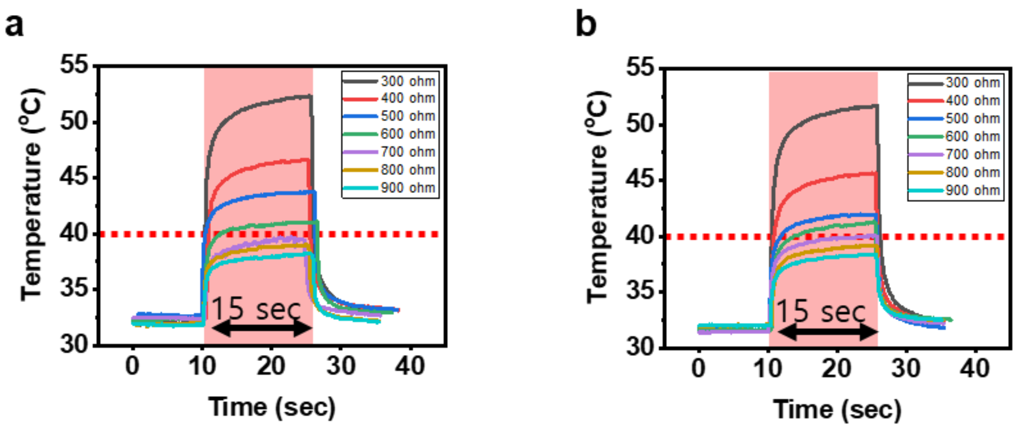

Figure S6. Temperature changes of various thermal actuators on Sylgard 170 (a) without encapsulation and (b) with encapsulation.

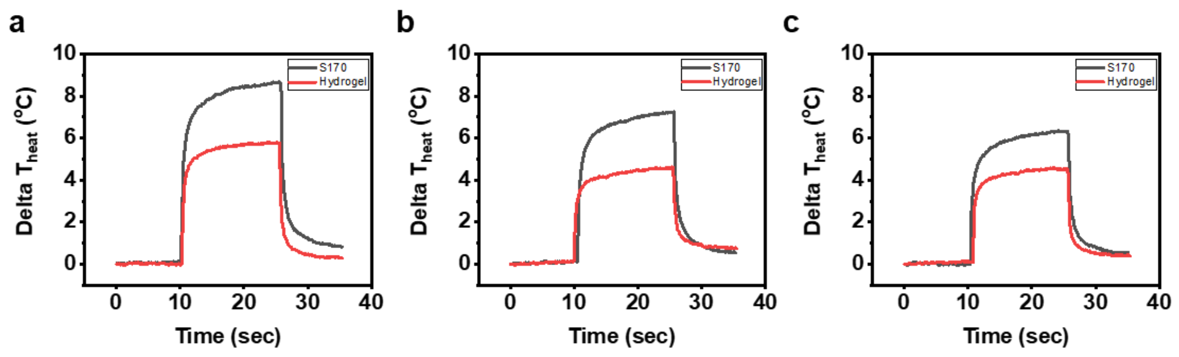

Figure S7. Temperature changes measured on S170 and hydrogel using resistors with resistances of (a) 700 Ω, (b) 800 Ω, and (c) 900 Ω.

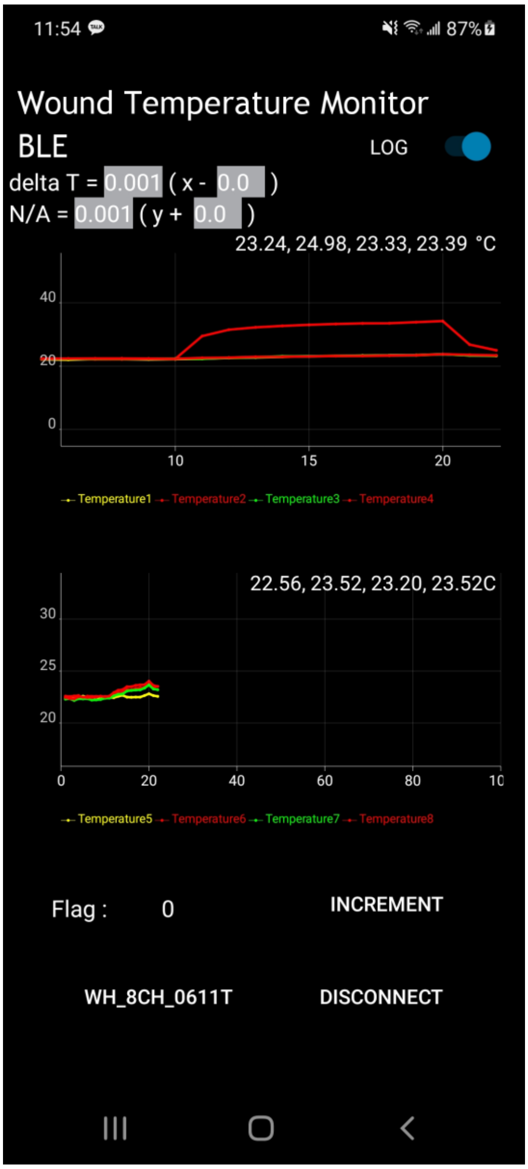

Figure S8. Screen shot of the smartphone user interface for the wound temperature monitoring system.

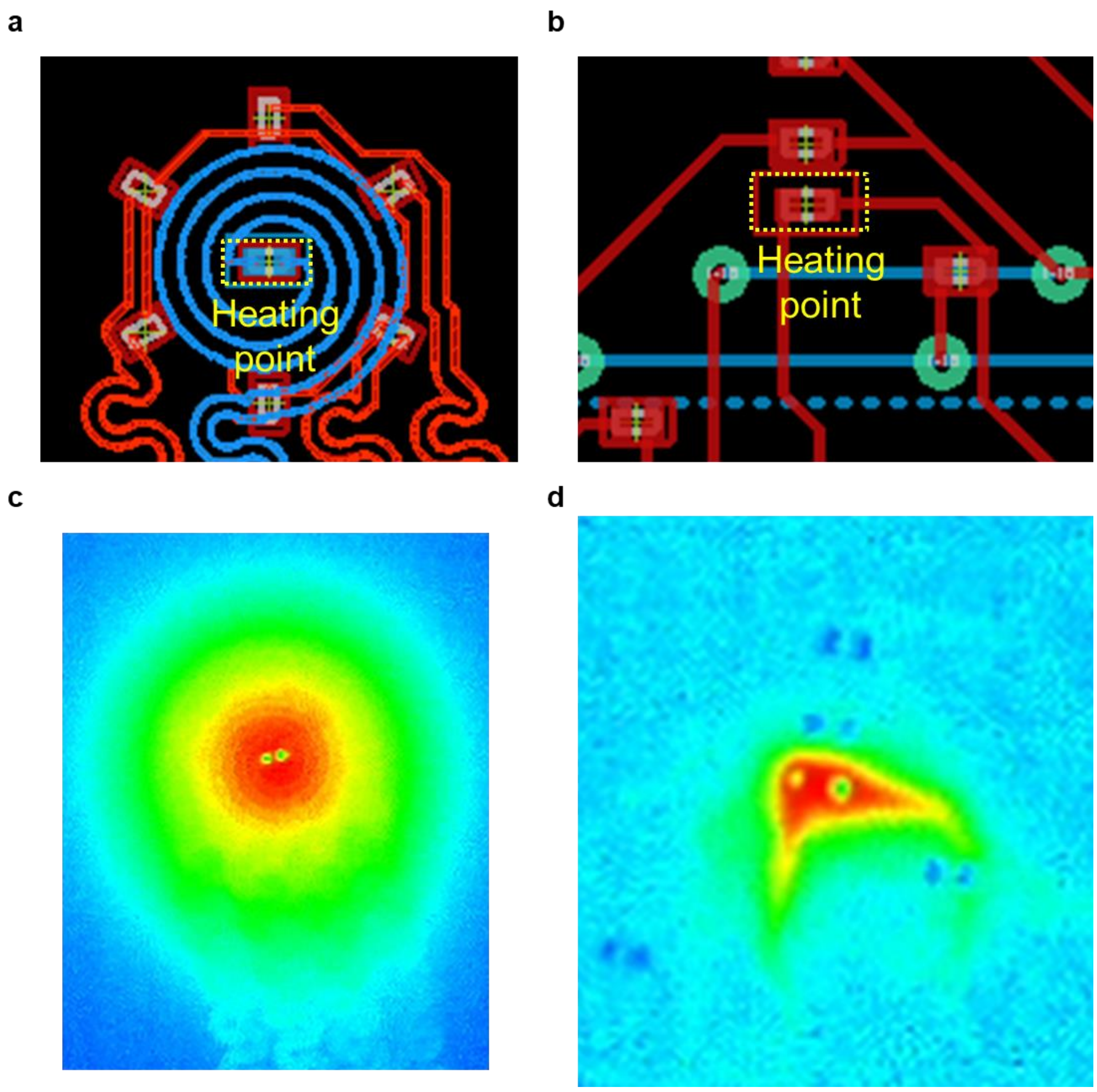

Figure S9. (a) Spiral and (b) linear interconnect designs of printed circuit boards. Heat distribution for the (c) spiral and (d) linear interconnect designs.

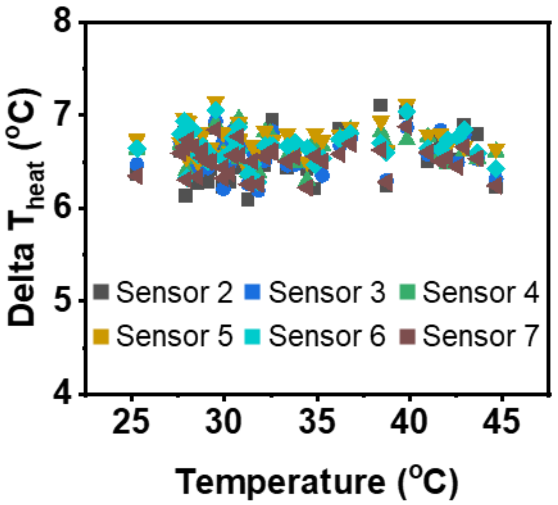

Figure S10. Maximum ΔT_{heat} of sensor 2-7 at various temperatures.

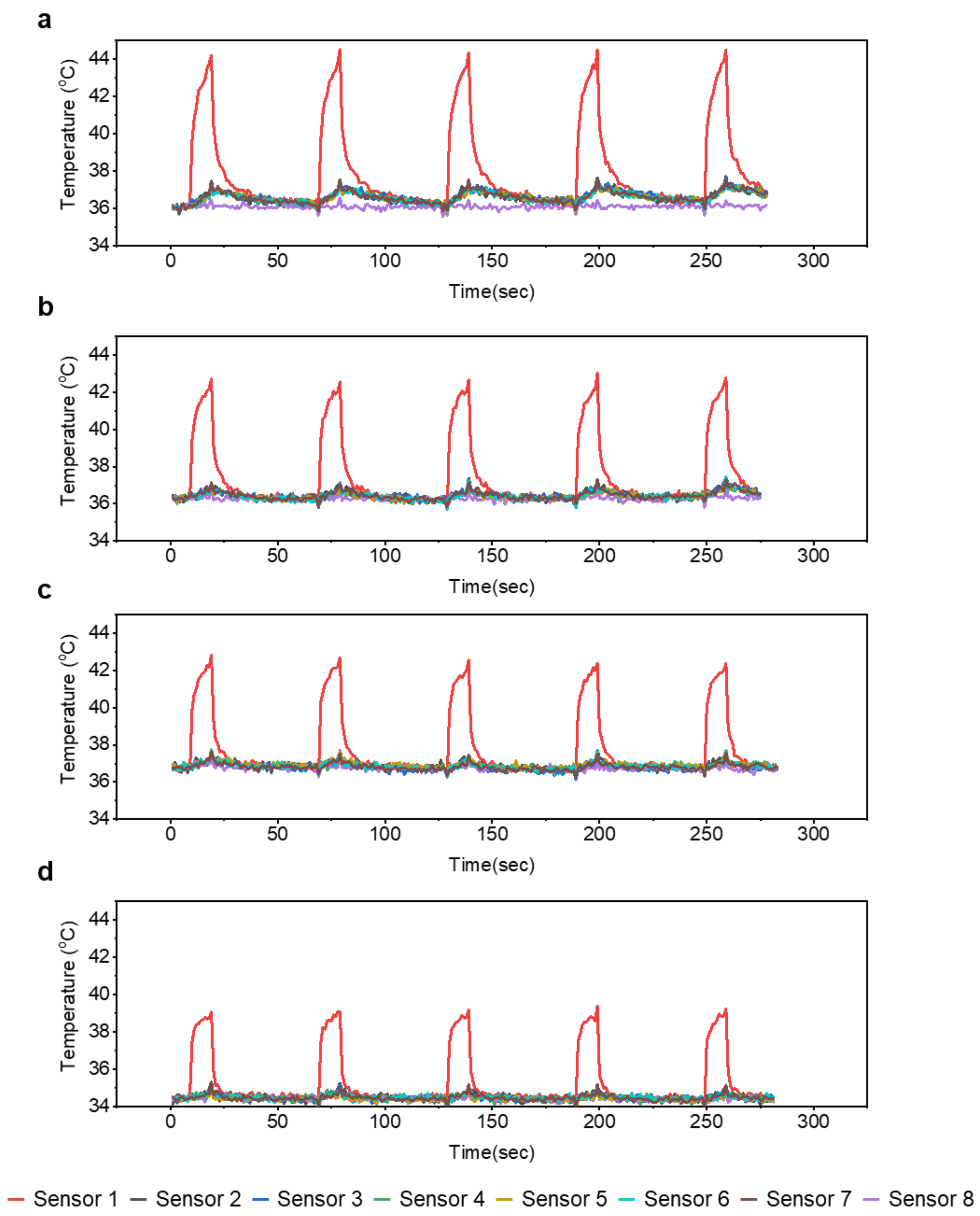

Figure S11. Wireless temperature measurement results in (a) air, (b) on Sylgard S184, (c) on Sylgard S170, and (d) in DPBS (pH 7.4). Measurements were performed in an oven.

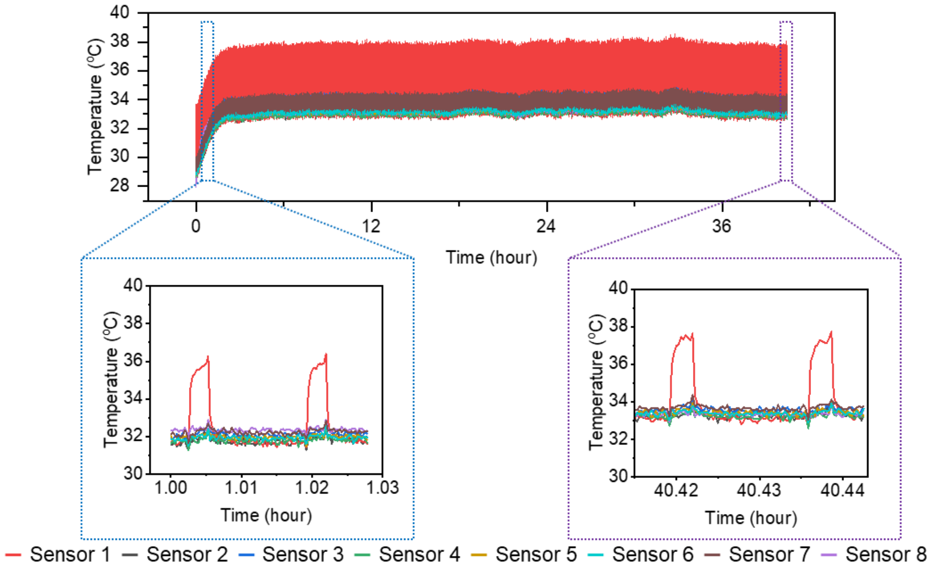

Figure S12. Long-term operation of the device during immersion in DPBS (pH 7.4).

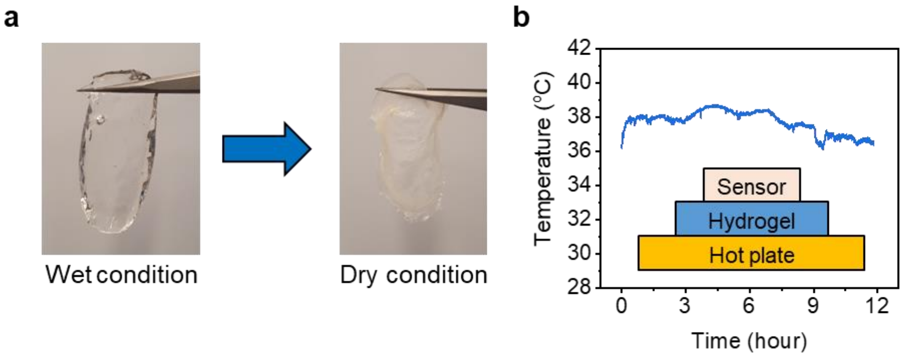

Figure S13. Temperature monitoring results on hydrogel. (a) Photographs of the hydrogel before and after drying. (b) Temperature changes measured on the hydrogel.

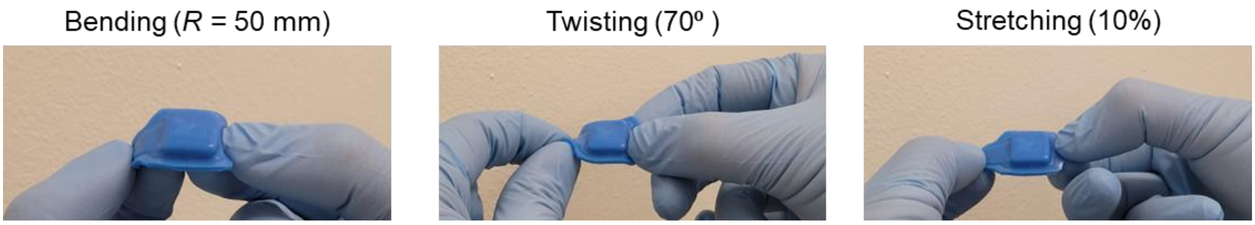

Figure S14. Photographs of the device during deformation by bending, twisting, and stretching.

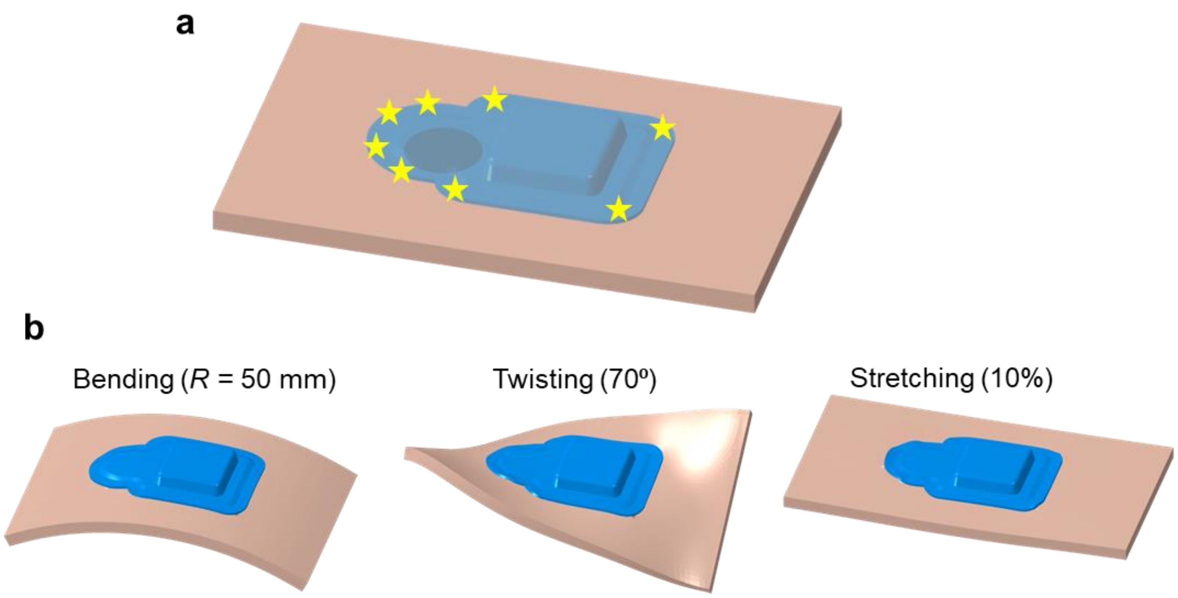

Figure S15. (a) Location of the undeformed WMS device relative to the wound site. Yellow stars indicate the suture locations. (b) Deformed configurations of the WMS during bending (radius of curvature 50 mm), twisting (70º), and uniaxial stretching (10%), respectively.

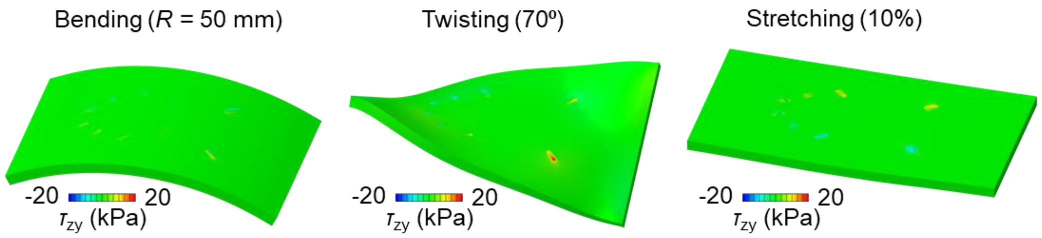

Figure S16. Computed shear stress (τ_{zy}) at the device/skin interface during bending (radius of curvature 50 mm), twisting (70º), and uniaxial stretching (10%), respectively. The interfacial shear stress (τ_{zy}) on the skin remains below the sensory perception threshold of 20 kPa for human skin.

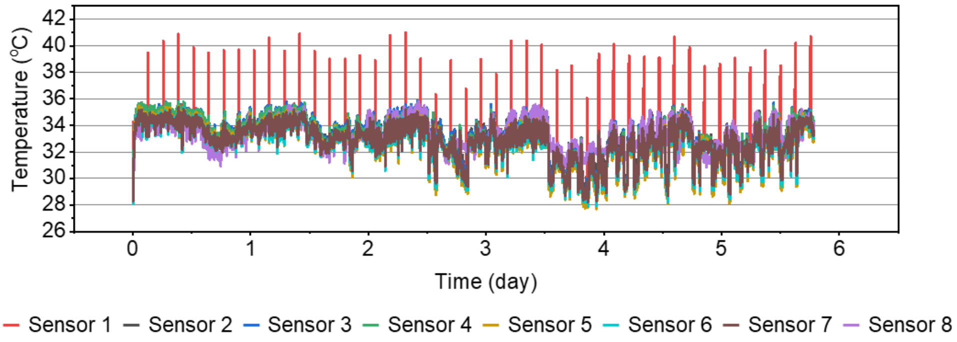

Figure S17. *In vivo* wireless temperature monitoring results obtained from a normal mouse (control group).

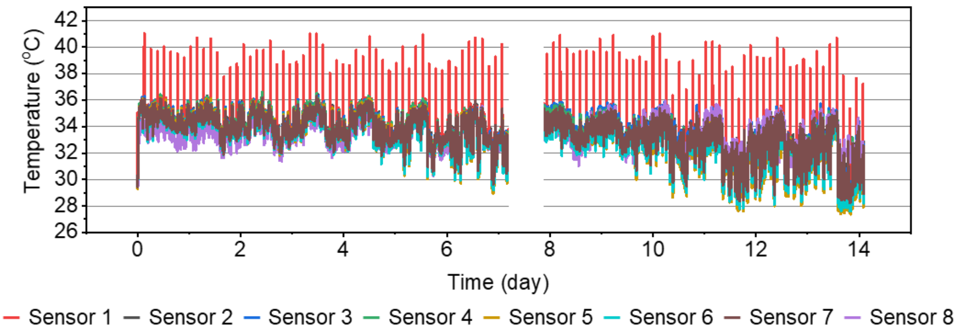

Figure S18. *In vivo* wireless temperature monitoring results obtained from a normal mouse (experimental group).

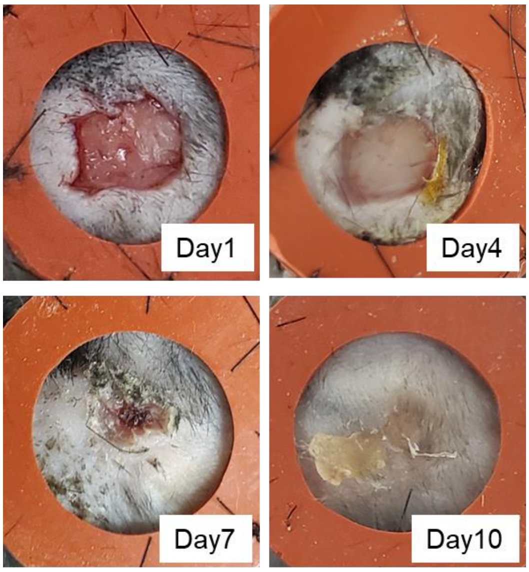

Figure S19. Photographs of the wound site of a normal mouse at various time points during the healing process.

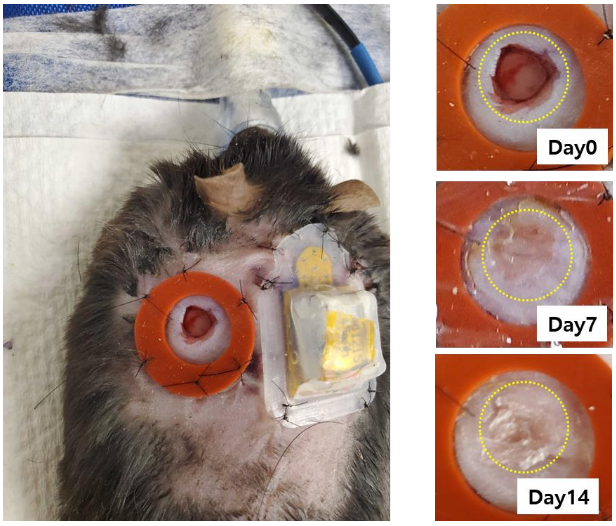

Figure S20. Photographs of the experimental configuratoin (left) and the wound healing process obtained using a diabetic mouse (right).

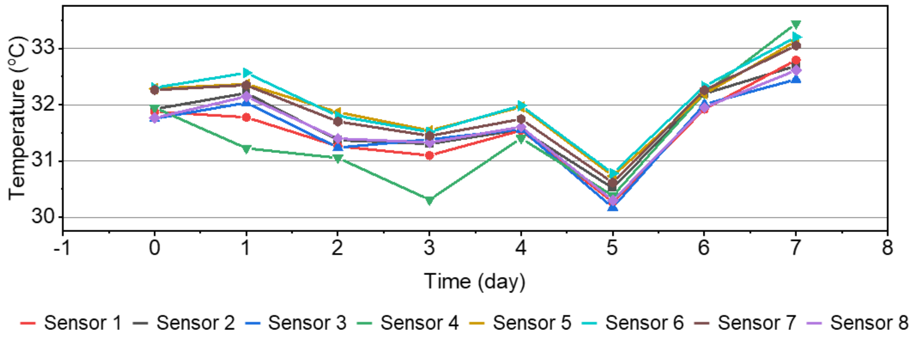

Figure S21. Wireless wound monitoring results obtained from a diabetic mouse using the NFC platform.

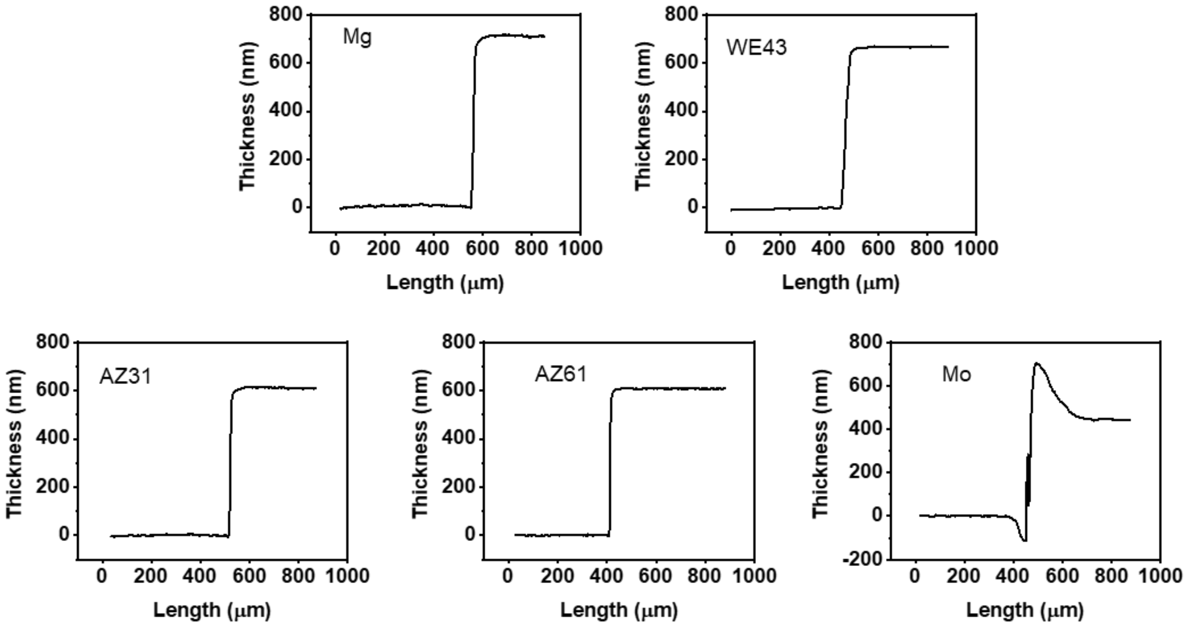

Figure S22. Thickness profiles of bioresorbable metal films deposited by electron beam evaporation.

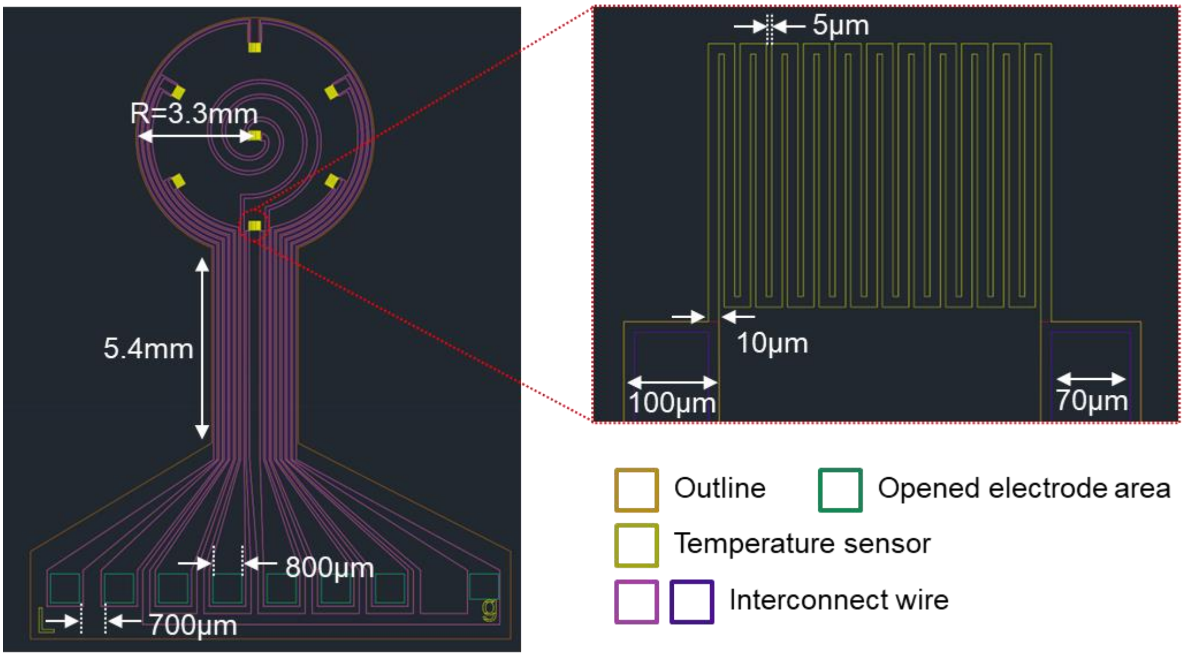

Figure S23. Design of a bioresorbable temperature sensor system.

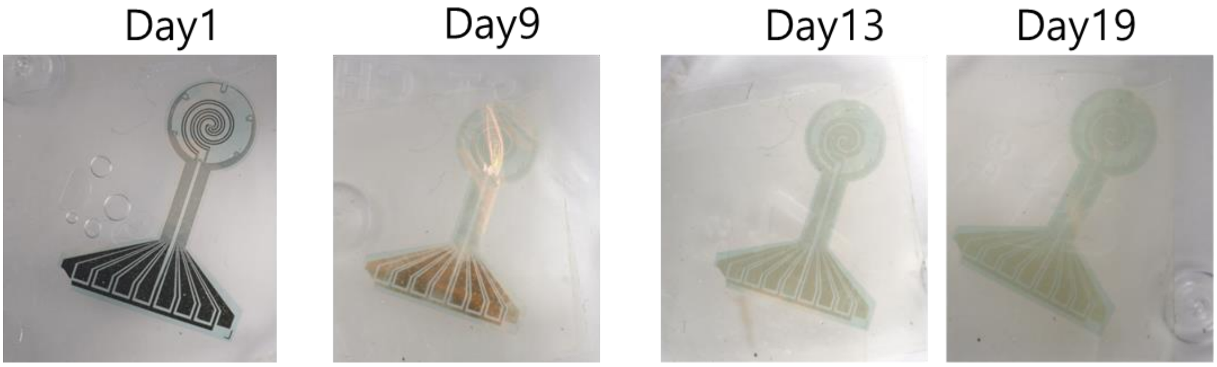

Figure S24. Photographs of dissolution of a bioresorbable sensor captured at different times of immersion in DPBS (pH 7.4) at 75 °C.

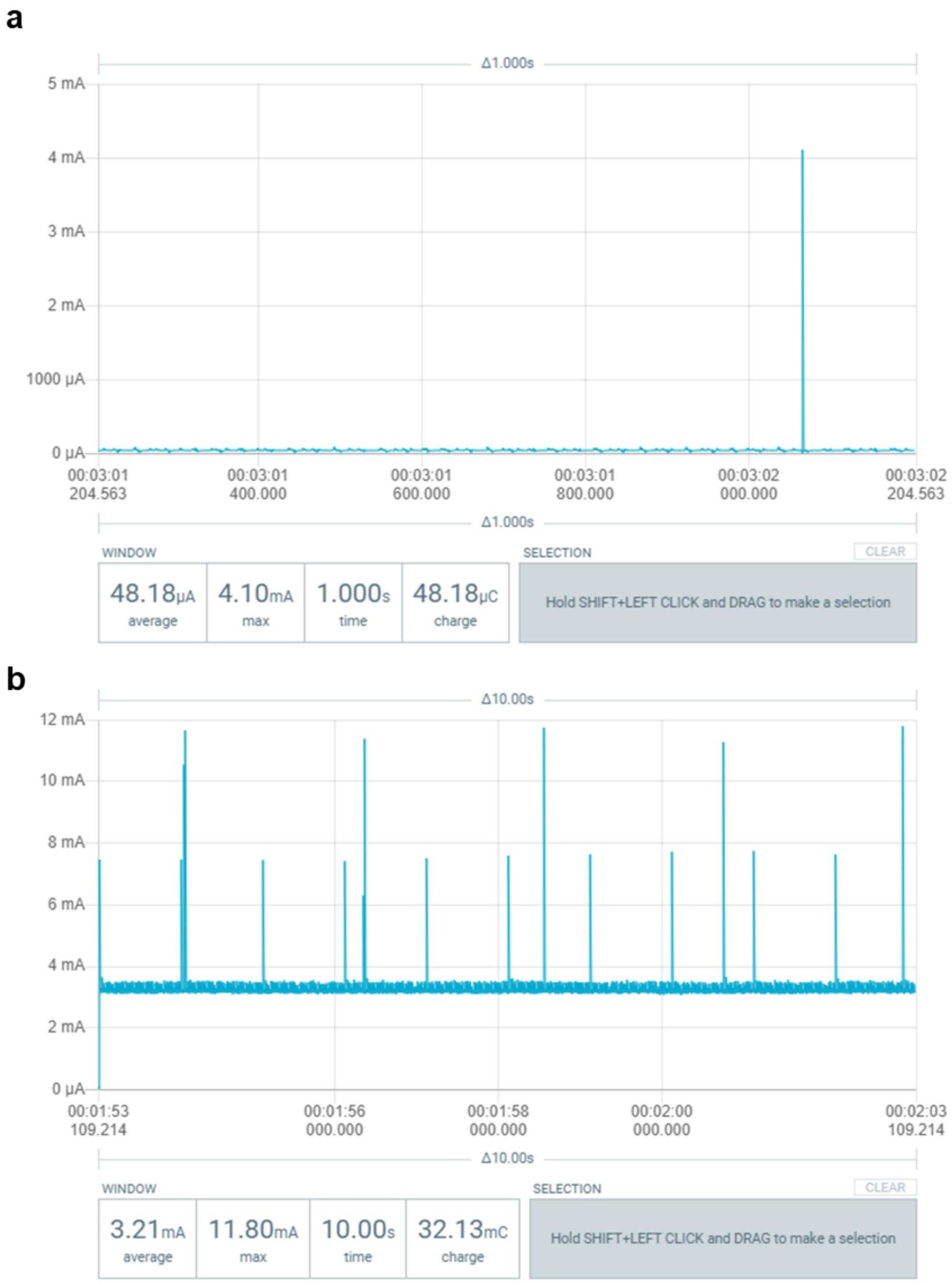

Figure S25. Power consumption of the WMS when (a) the temperature sensors activate and (b) the thermal actuator and temperature sensors activate together.

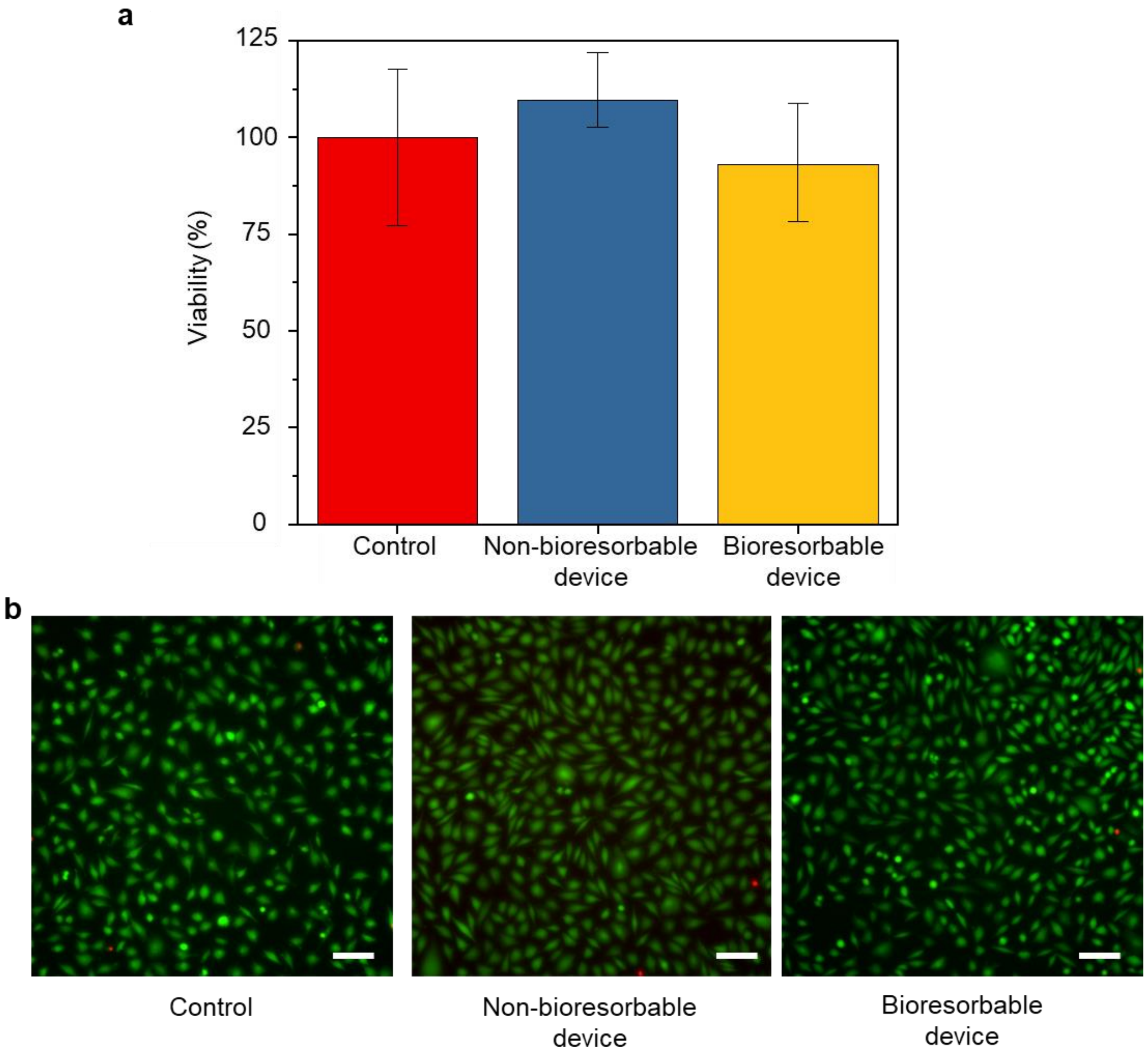

Figure S26. (a) Normalized in vitro viability assay of mouse fibroblasts (L929) after 96 hours of culture in tissue culture plate for the control, non-bioresorbable device (Silbion RTV 4420) and the bioresorbable device (data presented as mean ± S.D., n=3) (b) Live/Dead staining assay data (scale bar= 100µm).
